# Feeding Fiber-Bound Polyphenol Ingredients at Different Levels Modulates Colonic Postbiotics to Improve Gut Health in Cats

**DOI:** 10.3390/ani12131654

**Published:** 2022-06-27

**Authors:** Dennis E. Jewell, Matthew I. Jackson, Chun-Yen Cochrane, Dayakar V. Badri

**Affiliations:** 1 Department of Grain Science and Industry, Kansas State University, Manhattan, KS 66506, USA; djewell@ksu.edu; 2 Hill’s Pet Nutrition, Inc., Topeka, KS 66617, USA; matthew_jackson@hillspet.com (M.I.J.); chun-yen_cochrane@hillspet.com (C.-Y.C.)

**Keywords:** anti-inflammatory, bioactive metabolite, feline, fiber, microbiota, short-chain fatty acid, polyphenol

## Abstract

**Simple Summary:**

Food eaten by humans or companion animals is broken down by enzymes produced by the host and also by bacteria present in the large intestine of the host. Many of the compounds produced can have beneficial effects on the host’s health. Previous studies in dogs evaluated changes after they ate food containing a fiber bundle made of pecan shells, flax seed, and powders from cranberry, citrus, and beet. These studies showed that bacteria in the large intestine switched from digesting mainly protein to digesting mainly carbohydrates resulting in production of compounds with beneficial properties. The study presented here tested this fiber bundle in cats to see which compounds and/or bacteria in the feces changed. After cats consumed food containing the fiber bundle, several compounds associated with beneficial health effects increased, and some compounds that indicate the breakdown of protein decreased. In contrast, little change in fecal bacteria was observed following consumption of food with the fiber bundle. Overall, these findings indicate that, similar to the dog studies, bacteria in the large intestine of cats were able to digest the fiber bundle to make compounds that may contribute to host health and also shifted to digestion of carbohydrates instead of protein.

**Abstract:**

Consumption of fiber in its different forms can result in positive health effects. Prior studies in dogs found that addition of a fiber bundle (composed of pecan shells, flax seed, and powders of cranberry, citrus, and beet) to food resulted in a shift in fecal bacterial metabolism from proteolysis to saccharolysis. The present study evaluated the changes in fecal metabolites and microbiota in healthy cats following the consumption of this fiber bundle. Following a 28-day pre-feed period, 56 healthy adult cats received food with none or one of three concentrations (0%, 1%, 2%, and 4%) of the fiber bundle for a 31-day period. In cats that consumed the 4% fiber bundle, levels of ammonium and fecal branched-chain fatty acids (BCFAs) decreased from baseline and compared with the other groups. Addition of any level of the fiber bundle resulted in increases in beneficial metabolites: polyphenols hesperidin, hesperetin, ponciretin, secoisolariciresinol diglucoside, secoisolariciresinol, and enterodiol. Little change in fecal microbiota was observed. Since higher levels of ammonia and BCFAs indicate putrefactive metabolism, the decreases in these with the 4% fiber bundle indicate a shift toward saccharolytic metabolism despite little change in the microbiota composition.

## 1. Introduction

The gut microbiome produces numerous metabolites that vary depending on the food intake, microbiome community composition, and underlying health status of the host. In turn, these bacterially derived metabolites, including short-chain fatty acids (SCFAs), polyphenol metabolites, and other postbiotic compounds are bioavailable to the host. Health benefits to the host via alterations in the production of postbiotics by the gut microbiota is one goal of nutrition-based interventions [1]. These interventions can possibly reduce future disease development in healthy hosts or normalize metabolic pathways that were shifted in disease states, via the production of gut microbiome-produced metabolites, as changes in the metabolome can appear before the onset of clinical symptoms [1].

Many studies have examined the composition of the gut microbiome and metabolome in disease states, but some have also characterized them in healthy animals and humans. While domesticated cats are obligate carnivores, their gut microbiome is similar to that of omnivores [2]. Fiber is often added to commercially available cat foods [1], and results from several studies indicate that the gut microbial fermentation of fiber is beneficial in cats [3]. The inclusion of dietary fiber has been shown to result in favorable health outcomes in cats with diabetes or obesity [4,5]. In addition, positive changes in fecal metabolites, including increased polyphenols and decreased uremic toxins, were observed with the addition of dietary fiber in cats that were healthy or had chronic kidney disease [6,7].

Many of the prior studies that examined the effect the addition of fiber to cat food on the gut microbiome and/or metabolome supplemented food with cellulose and/or fructooligosaccharides [2,4,5,6,7,8,9], while few papers have reported the effects on cats of added dietary fiber-bound polyphenols. However, two prior studies tested the effects of the addition of a fiber bundle composed of pecan shells, flax seed, and powders of cranberry, citrus, and beet to dog food over 4-week periods [10,11]. At higher levels (14% *w*/*w*), inclusion of the fiber bundle appeared to shift metabolism from proteolysis to saccharolysis, as indicated by significant changes in fecal metabolites and the greater abundance of saccharolytic bacteria [10]. When the fiber bundle was added at lower levels (1%, 2%, or 4% *w*/*w*), significant increases in straight-chain SCFAs were observed with the fiber bundle added to 4%, and concentrations of several polyphenols were significantly higher with addition of any level of the fiber bundle [11]. These results may indicate positive health effects since SCFAs show several benefits to the host such as serving as an energy source, maintaining the integrity of the intestinal barrier, contributing to whole-host metabolism via fueling of hepatic biosynthesis pathways, and serving as signaling molecules [12]. However, no changes in the fecal microbiota were observed [11].

In the present study, the effects of this fiber bundle were tested at a range of levels (1%, 2%, or 4% *w*/*w*) in a parallel experimental design in healthy cats in order to examine the effects of these fiber-bound polyphenolic ingredients on fecal metabolites and microbiota. Serum biochemistry parameters, fecal SCFAs, selected fecal metabolites, and fecal microbiota are reported herein.

## 2. Materials and Methods

### 2.1. Study Foods

The study foods were produced at the Hill’s experimental food laboratory. The control food in this study was a complete and balanced food formulated to meet all the requirements of adult cats. The test foods were similar to the control food except for the addition of the fiber bundle (ground pecan shells, whole brown flax seed, beet pulp, citrus pulp, and cranberry pomace) at 1%, 2%, or 4% *w*/*w* on a dry matter basis that preserved the macronutrient composition of the control food (Table 1) by balancing the inclusion of the fiber bundle with barley, chicken, and chicken fat to account for the flax-derived protein and fat (Table S1). Comparison of macronutrients shows that the four foods are similar in moisture, crude protein, crude fat, ash, calcium, phosphorus, sodium, and several lipids (Table 1). All foods met the Association of American Feed Control Officials maintenance nutrition recommendations. Total polyphenols, including free and bound forms, were measured in the fiber bundle using the Folin–Ciocalteu method [13] and reported as gallic acid equivalents per gram extracted material. Briefly, free polyphenols were extracted using 80% acetone and bound polyphenols were extracted by the alkaline hydrolysis method. Based on the individual ingredient polyphenol measurements in the fiber bundle, polyphenol intakes were calculated as free, bound, and total for the foods used in this study.

### 2.2. Animals and Experimental Design

The study protocol was approved by the Hill’s Institutional Animal Care and Use Committee (IACUC; CP852a.0.0.0-A-F-D-ADH-MULTI-98-GI) and followed the US National Research Council’s guide for the care and use of laboratory animals [14].

Fifty-six healthy adult cats (27 female, 29 male), all spayed or neutered and owned by Hill’s Pet Nutrition, Inc. (Topeka, KS, USA), were included in this study. The cats were required to have no prior disease conditions (e.g., chronic gastrointestinal disease, renal disease) and had not received antibiotic interventions for at least one month. Cats with a history of food allergy or poor eating behavior were excluded from the study. All were housed with access to unrestricted socialization.

**Figure 1:**
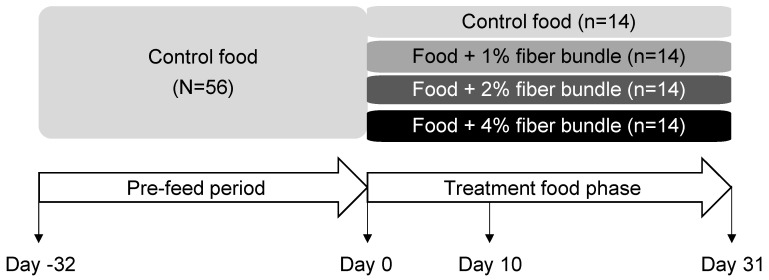
Study design and timeline in which cats consumed foods containing 0%, 1%, 2%, or 4% of the fiber bundle for 31 days. Blood and fecal samples were collected at days 0, 10, and 31 of the treatment food phase.

All cats were fed the control food for 32 days and then were divided into four groups of 14 based on their sex, age, and body weight to consume the control food or foods with the fiber bundle added to 1%, 2%, or 4% for 31 days (Figure 1). Feeding each cat the control food in the pre-feed phase prior to the treatment food phase was intended to reduce individual effects in the microbiome analysis. Every cat was fed based on caloric requirements as calculated from body weight. Food was available for 23 h/d. All cats were healthy at the end of the study and were returned to the colony with no adverse events reported.

Blood/serum and feces from each cat were collected at the end of the 32-day pre-feed (day 0) and at days 10 and 31 days in the treatment phase. Cats were sedated prior to phlebotomy. Blood was separated in serum separator tubes. Fecal samples were collected within 30 min following defecation and were obtained from 11 cats in the 4% fiber bundle group and 12 cats in each of the other groups.

### 2.3. Serum and Metabolite Analyses

Blood count profiles (Sysmex XN 1000-V, Sysmex America, Inc., Lincolnshire, IL, USA) and serum chemistry (Cobas c501, Roche Diagnostics, Indianapolis, IN, USA) were performed as in the manufacturers’ instructions. Fecal SCFAs and metabolites were analyzed by Metabolon, Inc. (Morrisville, NC, USA).

### 2.4. Stool Scoring and Fecal Sample Processing

Fecal scores were determined on a 1–5 scale (1, >75% liquid, no solid form; 2, 50% solid, 50% liquid, soft; 3, >75% formed and solid, some cylindrical shape; 4, >50% firm, >75% cylindrical; 5, >80% firm, cylindrical) [15]. Each fecal sample was homogenized in a Thinky Mixer (Thinky USA, Inc., Laguna Hills, CA, USA) via the Hill’s Pet Nutrition protocol [10], immediately followed by pH measurement and freezing at −70 °C until analysis.

### 2.5. Fecal Microbiome Analysis and Bioinformatics Processing

Fecal microbiome analysis was performed utilizing the Hill’s Pet Nutrition protocols [16] and as previously outlined [11]. Total DNA was extracted from fecal samples (PowerFecal DNA isolation kit, MO BIO, Carlsbad, CA, USA) prior to PCR amplification of the V3-V4 hypervariable regions of the 16S rRNA gene. The Illumina (San Diego, CA, USA) MiSeq platform was used for sequencing and de-multiplexing. Fast Adaptive Shrinkage Threshold Algorithm sequence files in text format associated with Quality scores (FASTQ) sequence files were processed via Mothur software [17], and the Greengenes reference database [18] was used for taxonomic classification. The Phylogenetic Investigation of Communities by Reconstruction of Unobserved States (PICRUSt) protocol [19] corrected for copy numbers of the 16S genes in operational taxonomic units (OTUs). Functional attributes were predicted using the Kyoto Encyclopedia of Genes and Genomes (KEGG) database [20].

### 2.6. Statistical Analysis

Microbiome data were analyzed at the phylum, family, and genus levels. Only the OTUs and the PICRUSt-predicted KEGG ortholog (KO) functions that passed the 70% prevalence cutoff in all of the fecal samples were considered for statistical analysis. The counts of individual phyla, families, and genera (corrected for 16S copy number) were analyzed by the negative binomial mixed models to test the effects of food treatments and collection timepoints (days 10 and 31). A principal coordinate analysis (PCoA) plot was made using the Manhattan distance of the relative abundances at the genus level at day 31. Permutational multivariate analysis of variance (PERMANOVA) based on the Manhattan distance of the relative abundances was used to compare beta diversity at the phylum, family, and genera levels as well as KEGG pathway functional compositions between food treatments. The PERMANOVA and all *p* values were adjusted for false discovery rate (FDR) by the Benjamini–Hochberg procedure.

For analysis of metabolites, values were natural log-transformed and the change was calculated by subtracting initial values from final values. The Proc Mixed procedure (SAS 9.4) was used to evaluate mean differences and change over time. Values are reported in relative fold differences, with significance noted for change from baseline (day 0) and differences among treatment groups.

## 3. Results

### 3.1. Study Design, Animals, and Food

Of the 56 healthy adult cats in this study, the mean ± SD age was 6.6 ± 3.0 years (range, 2.5–11.3 years). Mean body weight at baseline was 5.3 ± 1.0 kg (range, 3.1–7.7 kg; Table 2). All cats were fed the control food for 32 days in the pre-feed period prior to being divided into four groups (n = 14 each) that were fed the control food or test foods containing 1%, 2%, or 4% of the fiber bundle for 31 days. Food intake among the groups was similar. However, the intakes of free, bound, and total polyphenol content derived from the fiber bundle showed an increasing trend based on the fiber bundle inclusion level in the foods consumed by the cats in this study (Table S2). For example, inclusion of the 4% fiber bundle showed higher intake levels (mg/BW^0.75^) of free (35.8 ± 5.01), bound (43.7 ± 6.12) and total (79.5 ± 11.1) polyphenols compared with the other study foods.

After 31 days of the food treatment period, slightly greater weight gain from baseline was observed in cats in the 2% and 4% fiber bundle groups (0.08 kg and 0.07 kg) but not in the control or 1% fiber bundle groups (Table 2). Significant decreases from baseline were observed for total protein and triglycerides in the control and 1% fiber bundle groups. In addition, all four food groups showed significant decreases from baseline in urea nitrogen and slight but significant increases from baseline in creatinine. Among food groups, cholesterol showed a significantly greater decrease from baseline in the 2% fiber bundle group (−15.1 mg/dL) compared with the other three groups (0.1, −2.1, and 6.9 mg/dL). Nevertheless, levels of all of these circulating markers were within clinically normal ranges.

### 3.2. Fecal Parameters and Metabolites

Fecal moisture significantly differed among some of the test food groups at baseline, day 10, and day 31, but these changes were relatively minor and were not considered to be physiologically relevant (Table 3). Levels of fecal ammonium also somewhat differed among groups, but the 4% fiber bundle group was the only one to show a significant within-group decrease from baseline at day 31. Fecal pH was similar among all test food groups, with significant within-group changes from baseline at day 10 in the control and 4% fiber bundle groups, but all groups were similar to baseline by day 31. Stool firmness scores were at acceptable levels throughout the study and showed no significant differences between treatments or time points.

Some increases in some of the straight-chain SCFAs from baseline to Days 10 and/or 31 were seen, but no clear pattern emerged (Table 4). For example, propionic acid was significantly higher from baseline at day 10 but not day 31 in the 4% fiber bundle group. Similarly, butyric acid was significantly greater from baseline at only day 10 in the control group. Levels of butyric acid were significantly higher from baseline at both timepoints in the 1% fiber bundle group, but not in the 2% or 4% fiber bundle groups. Valeric acid and hexanoic acid were significantly greater than baseline at day 31 only in the 2% fiber bundle group.

In contrast, significant decreases from baseline to day 31 were observed for the branched-chain SCFAs 2-methylpropionic acid, 2-methylbutyric acid, and 3-methylbutyric acid in the 4% fiber bundle group (Table 4). In addition, these were all significantly different among the test food groups. A few other significant changes from baseline were noted, including increases at day 10 in the control food group for 2-methylbutryic acid and 3-methylbutryic acid. The 1% fiber bundle group also showed significant increases from baseline in 3-methylbutryic acid at days 10 and 31.

Significantly increased fecal levels of several polyphenols were observed with inclusion of the fiber bundle at 1%, 2%, or 4% (Table 5). Hesperidin, hesperetin, ponciretin, secoisolariciresinol diglucoside (SDG), secoisolariciresinol, and enterodiol were all greater than baseline at days 10 and 31 for all fiber bundle groups with the exception of no significant change in SDG in the 1% fiber bundle group at either timepoint. The levels of each of these metabolites also increased significantly with increased levels of the fiber bundle. Fecal levels of arabinose and ribulose/xylulose were significantly increased from baseline in both the 2% and 4% fiber bundle groups at day 31. In contrast, the control food group exhibited significant decreases from baseline in arabinose and ribulose/xylulose at both timepoints or day 10, respectively.

### 3.3. Fecal Microbiota

**Figure 2:**
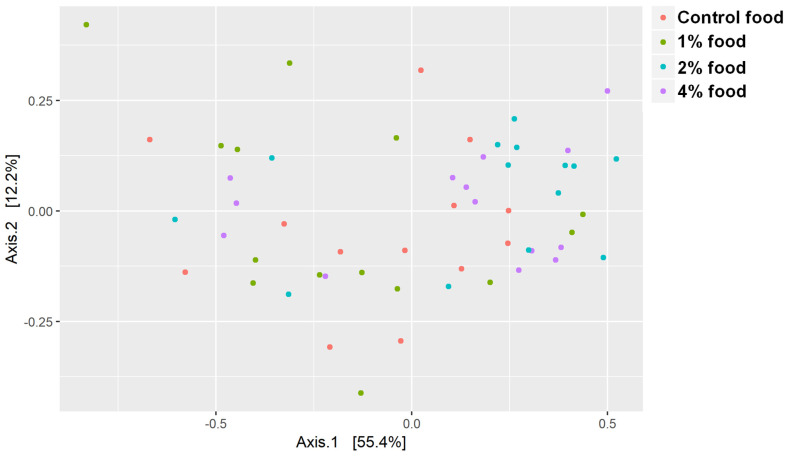
Principal coordinate analysis of the fecal microbiome of cats that consumed foods containing 0% (blue), 1% (red), 2% (green), or 4% (purple) of added fiber-bound polyphenol ingredients for 31 days.

**Figure 3:**
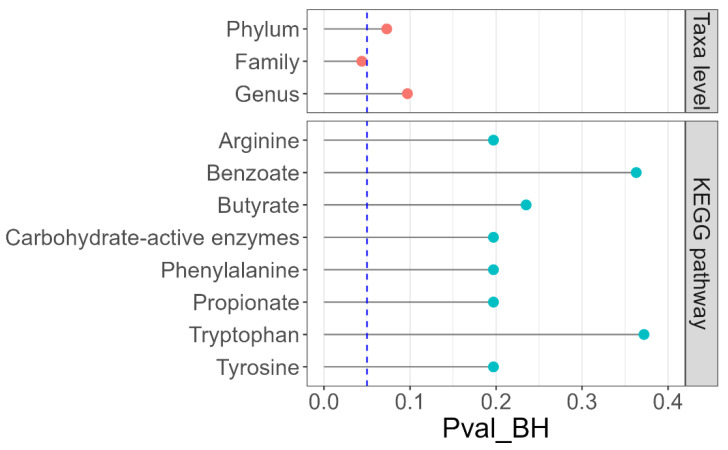
PERMANOVA analyses showing FDR-corrected *p* values of bacterial taxa (genus, family, and phylum level) and KEGG pathways in feces from cats that consumed the study foods. Vertical dotted line represents *p* = 0.05. KEGG, Kyoto Encyclopedia of Genes and Genomes; PERMANOVA, permutational multivariate analysis of variance; Pval_BH, *p* value corrected by the Benjamini–Hochberg method.

The PCoA plot showed no separation among any of the food types in the fecal microbiome (Figure 2). In addition, PERMANOVA analysis of the fecal microbiome composition at the phylum, family, and genera levels showed only a significant difference at the family level (*p* = 0.044; Figure 3), but no clear pattern emerged when examining individual family abundances among the treatments. None of the examined KEGG pathways showed significant differences in their compositions after multiple test corrections using the Benjamini–Hochberg method. However, the propionate pathway and enzymes involved in the modification and breakdown of polysaccharides (carbohydrate active enzymes) showed significant differences between treatments before multiple test correction.

## 4. Discussion

As observed in the studies in dogs [10,11], consumption by cats of a fiber bundle with fermentable fibers (hemicellulose, pectin), moderately fermentable fibers (rhamnogalacturonan, arabinoxylan polysaccharides) [21], insoluble bulking fiber (lignin) along with polyphenols (flavanones, flavonols, lignans) at levels of at least 4% (*w*/*w*) appeared to shift fecal microbial metabolism away from proteolysis and toward saccharolysis as indicated by decreased branched-chain fatty acids (BCFAs) and ammonium along with increased presence of monosaccharides derived from indigestible polysaccharide fibers. In addition, increased levels of fecal metabolites, including polyphenols and lignans with known beneficial health effects, were seen when the fiber bundle was consumed at any of the levels tested in cats.

Although the results of studies of this fiber bundle in cats and dogs [10,11] all indicate a shift toward saccharolytic metabolism in the fecal microbiota, the mechanisms appear to be different. Here, fecal ammonium significantly decreased (by 14%) at day 31 compared with baseline levels in the 4% fiber bundle group, and a decrease in fecal ammonia with consumption of resistant starch has also been observed in humans [22]. In contrast, ammonium was relatively unchanged in feces from dogs that consumed the fiber bundle [11]. In the dog study testing the fiber bundle at 1–4%, the only changes observed in SCFAs were significant increases from baseline at day 31 in the straight-chain SCFAs butyric acid, valeric acid, and hexanoic acid, all in the 4% fiber bundle group. In contrast, increases from baseline in straight-chain SCFAs in cats did not follow a clear pattern. However, levels of the BCFAs 2-methylpropionic acid, 2-methylbutyric acid, and 3-methylbutyric acid in cats were all significantly decreased from baseline at day 31 and were significantly different from the other test food groups in the 4% fiber bundle group. Increased ammonia and BCFAs are characteristic of putrefactive metabolism, indicating that consumption of the fiber bundle in cats at levels of at least 4% shifts the gut microbial metabolism away from a putrefactive state. This discrepancy between canine and feline responses to the fiber bundle may be associated with the different levels of dietary protein in their respective foods. Cats require higher dietary protein inclusion, perhaps leading to susceptibility towards fiber decreasing putrefaction of undigested protein in this species, whereas the higher dietary starch levels in dog foods may have enabled the increased saccharolysis observed in the previous study [11].

In contrast to the changes observed in SCFAs, the changes in selected fecal metabolites, including the polyphenols hesperidin, hesperetin, ponciretin, SDG, secoisolariciresinol, and enterodiol, were largely similar in both the dogs and cats that consumed food with the fiber bundle at 1–4%. Fiber-bound polyphenols arrive largely intact to the large intestine, where they are metabolized by intestinal microbiota [23]. Several lines of evidence indicate that food-derived polyphenols interact with the gut microbiota to benefit host health. Flavonoids are a class of polyphenols, and their consumption appears to lower the risk of cardiovascular diseases, cancer, metabolic diseases, and neurodegenerative diseases in humans [24,25]. Notably, three flavonoids found in or derived from citrus fruits (hesperidin, hesperetin, and ponciretin) were significantly increased in feces from cats that consumed any level of the fiber bundle in this study.

Hesperidin reduces inflammation via regulation of proinflammatory cytokines and decreases oxidative stress, thus conferring a variety of health benefits [26]. Supplementation with the citrus polyphenols hesperidin and naringin led to decreases in inflammatory markers produced by peripheral blood mononuclear cells in obese cats [27]. In the CITRUS trial in humans, consumption of orange juice supplemented with 600 mg hesperidin for 12 weeks appeared to result in the downregulation of six proinflammatory genes [28], decreased blood pressure [29], and lowered levels of several uremic toxins in the urine [30]. Other randomized, double-blind clinical trials showed that consumption of flavonoid-rich orange juice benefitted cognitive function in middle-aged [31] and older adults [32]. In rats fed an obesogenic diet, supplementation with hesperidin for 8 weeks resulted in decreased total cholesterol, blood pressure, and insulin sensitivity. Improvements were also observed in markers of inflammation, lipid profile, and metabolites related to oxidative stress [33]. Similar beneficial effects of hesperidin on inflammation, lipid profiles, oxidative stress, and hypertension were observed in humans [26,34]. Hesperidin also appears to have promising anticancer effects from in vitro experiments and in rodent models [35].

Hesperidin is metabolized to hesperetin by intestinal bacteria [36]. The higher bioavailability of hesperetin likely results in its greater anti-inflammatory and antioxidant abilities compared with hesperidin [26]. In fact, many of the in vivo effects observed with hesperidin supplementation may be due to its conversion to hesperetin. As with hesperidin, supplementation with hesperetin has been shown to lead to decreased inflammation and oxidative stress [37], effects that contribute to observed improvements in hypertension, metabolic disorder, and diabetes [34].

Both hesperidin and hesperetin have been shown to protect neurons against induced cytotoxicity in vitro and improve cognitive and motor impairments in animal models, including those for epilepsy, Huntington disease, Alzheimer disease, and Parkinson disease [24,38]. In addition, both metabolites appear to enhance gastrointestinal health by improving intestinal barrier integrity and colitis symptoms in vitro and in rodent models [25,39].

Ponciretin is a flavonoid metabolized by gut bacteria from its citrus-derived precursor poncirin [36,40]. Like the other citrus flavonoids, ponciretin also appears to have antiinflammatory [40,41] and anticancer [36] effects.

SDG is the predominant lignan in flaxseed and can be converted to secoisolariciresinol, enterodiol, and enterolactone by the gut microbiota [42,43,44]. Similar to this study and the ones testing the same fiber bundle in dogs [10,11], greater fecal levels of SDG and secoisolariciresinol were observed with increasing consumption of fiber [45]. Production of the postbiotic enterodiol is observed in a subset of humans [46]. The current study in cats as well as the preceding publications with canine subjects [10,11] provide evidence that both of these companion animal species, like some humans, harbor the gut microbes that lead to the enterodiol-producing phenotype. Like the citrus-derived polyphenols, SDG has anti-inflammatory [47,48] and antioxidant [49] properties and appears to have beneficial effects on several health states, including cancer, cardiovascular disease, and diabetes [42,43,50,51]. Both SDG and enterodiol have shown anticancer effects in vitro [50,51]. Long-term intake of lignans in people in the US was associated with a significantly lower risk of coronary heart disease, with a 13% risk reduction with secoisolariciresinol supplementation [52]. In addition, there may be a neuroprotective role for lignans. Patients with Alzheimer disease have lower levels of the neurotransmitter acetylcholine, which leads to memory loss, so it is hypothesized that dietary supplementation with lignans such as secoisolariciresinol, which can inhibit acetylcholinesterase, could offer neuroprotection [53].

In addition to the polyphenols, there were significant increases in fecal levels of arabinose and ribulose/xylulose following consumption of the fiber bundle at 2% or 4% in cats. These pentose sugars likely are the result of the metabolism of the fiber bundle, which included flax that contains arabinoxylan fibers [21], and are similar to the results observed in dogs that consumed the fiber bundle [11].

The relative lack of change in the gut microbiota in this study is not altogether surprising, given the prior results of lower levels of this fiber bundle in dogs [11] and the observations that dietary changes in the microbiota of healthy animals are often not as pronounced as disease-induced changes in microbiota [54]. However, the changes in fecal metabolites observed following consumption of the fiber bundle indicate that the existing gut microbiota were able to metabolize the fiber bundle. The composition of the gut microbiota did shift toward saccharolytic bacteria when the fiber bundle was included at levels of 14% (*w*/*w*) in dogs [10], so it is plausible that a shift in the gut microbiota may be observed in cats if the fiber bundle were included at levels greater than 4%.

This study may also have been limited by the relatively short 31-day feeding period with the fiber bundle, as additional changes in fecal metabolites and/or microbiota may be observed with a longer trial. A very long-term trial, though difficult to execute, would show whether consumption of the fiber bundle over months or years may result in a decreased risk of developing certain health conditions. In addition, consumption of the fiber bundle in cats with a health condition such as chronic kidney disease, diabetes, or gastroenteritis may result in more obvious changes and/or benefits to health. Some prior work has indicated that supplementation with other fibers leads to changes in the metabolome and microbiome in cats with chronic kidney disease and lowers serum glucose concentrations in cats with diabetes [4,6,55]. However, further investigation will be required to investigate how the specific fiber sources improve host health through impacting microbial metabolism on each targeted disease or condition.

## 5. Conclusions

In this study, a fiber bundle included at levels from 1–4% in cat food was evaluated for its effects on fecal metabolites and microbiota. Several fecal metabolites, including hesperidin, hesperetin, ponciretin, SDG, secoisolariciresinol, and enterodiol, all of which have established positive effects on health, were increased with addition of the fiber bundle at any level. Feces from cats that consumed the 4% fiber bundle exhibited decreases in ammonium and BCFAs, indicative of a shift away from proteolytic metabolism. There was little to no effect on the fecal microbiota following consumption of the fiber bundle at the levels tested. Overall, the results indicate that consumption of the fiber bundle by cats led to a shift toward saccharolytic metabolism by changing the physiology of the microbiota and transforming fiber-bound polyphenols into bioactive metabolites for beneficial health effects.

## Figures and Tables

**Table 1:** Composition of test foods containing 0, 1, 2, or 4% of microbial-targeted ingredients (grams/100 g as mixed or as fed, unless otherwise stated).

		Fiber Bundle Percentage in Food		
Nutrient Parameter	Control Food	1%	2%	4%
Moisture	4.97	4.70	4.76	4.30
Crude protein	37.00	36.63	35.81	38.06
Crude fat	19.53	19.31	19.34	19.67
Atwater energy ^1^ (kcal/kg)	4066	4049	4031	4052
Calories (kcal/kg)	5225	5225	5313	5357
Ash	6.23	6.09	6.41	6.33
Crude fiber	0.6	1.1	1.3	1.7
Nitrogen-free extract	31.7	32.2	32.4	29.9
Total dietary fiber	4.3	5.3	4.6	6.1
Total insoluble fiber	3.2	4.7	4.2	5.3
Total soluble fiber	1.1	0.6	0.4	0.8
Neutral detergent fiber	3.70	5.20	4.20	4.30
Calcium	1.09	1.09	1.18	1.15
Phosphorus	0.87	0.90	0.92	0.90
Sodium	0.33	0.36	0.34	0.34
Omega-3 sum	0.19	0.25	0.28	0.36
Omega-6 sum	3.41	3.55	3.40	3.60
Palmitic acid [16:0]	4.23	4.38	4.43	4.23
Palmitoleic acid [16:1]	1.08	1.13	1.09	1.15
Stearic acid [18:0]	1.08	1.10	1.09	1.15
Oleic acid [18:1]	6.78	7.03	6.82	7.19
Linoleic acid [18:2 (n − 6)]	3.15	3.29	3.15	3.31
Alpha-linolenic acid [18:3 (n − 3)]	0.14	0.20	0.23	0.31
Arachidonic acid [20:4 (n − 6)]	0.12	0.12	0.13	0.14
Lysine	2.01	2.01	2.03	2.34
Threonine	1.37	1.39	1.39	1.44
Methionine	1.29	1.27	1.25	1.35
Cystine	0.51	0.50	0.47	0.49
Tryptophan	0.36	0.35	0.36	0.40

^1^ Calculated from analytical values using modified Atwater numbers (kcal/g of 3.5 for protein, 8.5 for fat, and 3.5 for nitrogen-free extract).

**Table 2:** Body weight and selected serum biochemistry parameters at baseline (Day 0), end of study, and change from baseline in cats that consumed foods containing 0%, 1%, 2%, or 4% of added fiber-bound polyphenol ingredients.

		Fiber Bundle Percentage in Food		
Parameter	Control Food	1%	2%	4%
Body weight, kg				
Day 0	5.60 ± 0.26	5.60 ± 0.26	5.09 ± 0.26	5.10 ± 0.26
Day 31	5.63 ± 0.25	5.65 ± 0.25	5.17 ± 0.25	5.17 ± 0.25
Change	0.03 ± 0.03	0.05 ± 0.03	0.08 ± 0.03 ^1^	0.07 ± 0.03 ^1^
Food intake, kcal/(body weight in kg) ^0.75^	53.0 ± 2.5	55.8 ± 2.5	54.4 ± 2.5	53.5 ± 2.5
Albumin, mg/dL				
Day 0	3.34 ± 0.08	3.27 ± 0.08	3.29 ± 0.08	3.30 ± 0.08
Day 31	3.38 ± 0.08	3.29 ± 0.08	3.31 ± 0.08	3.38 ± 0.08
Change	0.03 ± 0.04	0.01 ± 0.04	0.01 ± 0.04	0.08 ± 0.04
Total protein, mg/dL				
Day 0	6.59 ± 0.12	6.73 ± 0.12	6.72 ± 0.12	6.55 ± 0.12
Day 31	6.39 ± 0.12	6.46 ± 0.12	6.57 ± 0.12	6.41 ± 0.12
Change	−0.20 ± 0.08 ^1^	−0.26 ± 0.08 ^1^	−0.15 ± 0.08	−0.14 ± 0.08
Urea nitrogen, mg/dL				
Day 0	22.6 ± 0.9	23.0 ± 0.9	22.5 ± 0.9	22.1 ± 0.9
Day 31	20.1 ± 0.9	20.5 ± 0.9	20.7 ± 0.9	20.8 ± 0.9
Change	−1.4 ± 0.4 ^1^	−2.5 ± 0.4 ^1^	−1.9 ± 0.4 ^1^	−1.3 ± 0.4 ^1^
Creatinine, mg/dL				
Day 0	1.15 ± 0.05	1.19 ± 0.05	1.11 ± 0.05	1.12 ± 0.05
Day 31	1.25 ± 0.05	1.28 ± 0.05	1.18 ± 0.05	1.22 ± 0.05
Change	0.10 ± 0.03 ^1^	0.10 ± 0.03 ^1^	0.07 ± 0.03 ^1^	0.10 ± 0.03 ^1^
Triglycerides, mg/dL				
Day 0	54.4 ± 9.5	54.6 ± 9.5	54.0 ± 9.5	59.8 ± 9.5
Day 31	36.1 ± 10.7	41.4 ± 10.7	41.6 ± 10.7	57.6 ± 10.7
Change	−18.2 ± 7.5 ^1^	−15.1 ± 7.5 ^1^	−12.4 ± 7.5	−2.2 ± 7.5
Cholesterol, mg/dL				
Day 0	212.9 ± 15.0	208.0 ± 15.0	203.0 ± 15.0	220.6 ± 15.0
Day 31	213.0 ± 15.3	205.9 ± 15.3	187.9 ± 15.3	227.4 ± 15.3
Change	0.1 ± 4.5 ^a^	−2.1 ± 4.5 ^a^	−15.1 ± 4.5 ^b,1^	6.9 ± 4.5 ^a^

Values are least square means ± standard errors. ^1^ Significantly different (*p* < 0.05) from baseline (Day 0). Different superscripted letters represent significant differences within a row (*p* < 0.05).

**Table 3:** Fecal moisture, ammonium, and pH at baseline (Day 0), end of study, and change from baseline and stool scores in cats that consumed foods containing 0%, 1%, 2%, or 4% of added fiber-bound polyphenol ingredients.

		Fiber Bundle Percentage in Food		
Parameter	Control Food	1%	2%	4%
Moisture				
Day 0	60.0 ± 0.7 ^a^	56.9 ± 0.9 ^b^	62.7 ± 0.7 ^c^	62.9 ± 0.8 ^c^
Day 10, % of Day 0	102 ± 2 ^b^	109 ± 2 ^a^	97 ± 2 ^b^	99 ± 2 ^b^
Day 31, % of Day 0	101 ± 2 ^a,b^	105 ± 2 ^a^	98 ± 2 ^b^	100 ± 2 ^a,b^
Ammonium, mmol/g				
Day 0	0.050 ± 0.002 ^a^	0.056 ± 0.002 ^b^	0.045 ± 0.002 ^c^	0.047 ± 0.002 ^a,c^
Day 10, % of Day 0	113 ± 5 ^a^	91 ± 5 ^b^	107 ± 5 ^a^	92 ± 5 ^b^
Day 31, % of Day 0	100 ± 5	93 ± 5	94 ± 5	86 ± 5 ^1^
pH				
Day 0	6.04 ± 0.07	6.04 ± 0.07	5.99 ± 0.07	5.97 ± 0.08
Day 10, % of Day 0	94 ± 2 ^1^	96 ± 2	93 ± 2	94 ± 2 ^1^
Day 31, % of Day 0	98 ± 2	98 ± 2	94 ± 2	96 ± 2
Stool score				
Day 0	4.91 ± 0.12	5.0 ± 0.12	4.66 ± 0.12	5.0 ± 0.12
Day 10	5.0 ± 0.12	4.91 ± 0.12	5.0 ± 0.12	4.63 ± 0.12
Day 31	4.91 ± 0.12	5.0 ± 0.12	4.75 ± 0.12	4.9 ± 0.12

Values are least square means ± standard errors. ^1^ Significantly different (*p* < 0.05) from baseline (Day 0). Different superscripted letters represent significant differences within a row (*p* < 0.05).

**Table 4:** Fecal short-chain fatty acids at baseline (Day 0) and days 10 and 31 in cats that consumed foods containing 0%, 1%, 2%, or 4% of added fiber-bound polyphenol ingredients.

		Fiber Bundle Percentage in Food		
SCFA	Control Food	1%	2%	4%
Acetic acid				
Day 0, μg/g	3428 ± 374	3273 ± 374	4106 ± 360	3895 ± 389
Day 10, % of Day 0	91 ± 6	107 ± 6	99 ± 6	111 ± 6
Day 31, % of Day 0	100 ± 6	110 ± 6	98 ± 6	107 ± 6
Propionic acid				
Day 0, μg/g	1741 ± 271	1762 ± 282	2138 ± 282	1953 ± 282
Day 10, % of Day 0	104 ± 7 ^a,b^	115 ± 8 ^a,b^	101 ± 8 ^a^	124 ± 8 ^b,1^
Day 31, % of Day 0	92 ± 7	97 ± 7	97 ± 7	111 ± 8
Butyric acid				
Day 0, μg/g	2254 ± 282	2216 ± 282	2406 ± 272	2204 ± 294
Day 10, % of Day 0	139 ± 9 ^a,1^	127 ± 8 ^a,b,1^	109 ± 9 ^b,c^	99 ± 9 ^c^
Day 31, % of Day 0	111 ± 9 ^a,b^	119 ± 9 ^a,1^	104 ± 9 ^a,b^	90 ± 9 ^b^
Valeric acid				
Day 0, μg/g	1270 ± 161	1234 ± 167	1148 ± 167	1266 ± 167
Day 10, % of initial	147 ± 129	151 ± 135	123 ± 135	151 ± 135
Day 31, % of initial	119 ± 129 ^b^	128 ± 129 ^b^	436 ± 125 ^a,1^	94 ± 135 ^b^
Hexanoic acid				
Day 0, μg/g	183 ± 39	258 ± 39	113 ± 38	197 ± 41
Day 10, % of Day 0	199 ± 87	239 ± 92	104 ± 92	258 ± 91
Day 31, % of Day 0	125 ± 87 ^b^	197 ± 88 ^a,b^	395 ± 85 ^a,1^	130 ± 91 ^b^
**BCFA**				
2-methylpropionic acid				
Day 0, μg/g	301 ± 21	314 ± 22	244 ± 22	250 ± 22
Day 10, % of Day 0	116 ± 10	110 ± 11	95 ± 11	93 ± 10
Day 31, % of Day 0	86 ± 11 ^a^	114 ± 10 ^b^	118 ± 10 ^b^	76 ± 11 ^a,1^
2-methylbutyric acid				
Day 0, μg/g	244 ± 19	276 ± 19	194 ± 19	199 ± 19
Day 10, % of Day 0	124 ± 10 ^1^	114 ± 10	110 ± 10	98 ± 10
Day 31, % of Day 0	87 ± 10 ^a^	115 ± 10 ^b^	116 ± 10 ^b^	74 ± 10 ^a,1^
3-methylbutyric acid				
Day 0, μg/g	357 ± 26	394 ± 27	304 ± 27	313 ± 27
Day 10, % of Day 0	125 ± 11 ^1^	124 ± 12 ^1^	101 ± 12	103 ± 12
Day 31, % of Day 0	92 ± 11 ^a^	125 ± 12 ^b,1^	119 ± 11 ^b^	77 ± 12 ^c,1^

^1^ Significantly different (*p* < 0.05) from baseline (Day 0). Different superscripted letters represent significant differences within a row (*p* < 0.05). BCFA, branched-chain fatty acid; SCFA, straight-chain fatty acid.

**Table 5:** Change from initial concentrations at days 10 and 31 (natural log day 10 or 31—natural log Day 0) of selected fecal metabolites in cats that consumed foods containing 0%, 1%, 2%, or 4% of added fiber-bound polyphenol ingredients.

		Fiber Bundle Percentage in Food		
Metabolite	Control Food	1%	2%	4%
Hesperidin				
Day 10 ratio	0.04 ± 0.25 ^a^	2.89 ± 0.26 ^b,1^	3.89 ± 0.26 ^c,1^	5.02 ± 0.26 ^d,1^
Day 31 ratio	0.05 ± 0.26 ^a^	2.22 ± 0.25 ^b,1^	3.85 ± 0.24 ^c,1^	4.91 ± 0.27 ^d,1^
Hesperetin				
Day 10 ratio	0.62 ± 0.38 ^a^	4.66 ± 0.17 ^b,1^	5.23 ± 0.17 ^c,1^	6.06 ± 0.17 ^d,1^
Day 31 ratio	0.67 ± 0.38 ^a^	4.19 ± 0.17 ^b,1^	5.12 ± 0.16 ^c,1^	5.94 ± 0.18 ^d,1^
Ponciretin				
Day 10 ratio	0.25 ± 0.14 ^a^	3.51 ± 0.14 ^b,1^	3.90 ± 0.14 ^c,1^	5.06 ± 0.14 ^b,1^
Day 31 ratio	0.12 ± 0.15 ^a^	3.21 ± 0.14 ^b,1^	4.06 ± 0.13 ^c,1^	4.86 ± 0.15 ^b,1^
Secoisolariciresinol diglucoside				
Day 10 ratio	0.00 ± 0.15 ^a^	0.27 ± 0.16 ^a^	0.46 ± 0.16 ^b,1^	1.14 ± 0.16 ^c,1^
Day 31 ratio	0.00 ± 0.16 ^a^	0.20 ± 0.15 ^a^	0.90 ± 0.15 ^b,1^	1.58 ± 0.17 ^c,1^
Secoisolariciresinol				
Day 10 ratio	0.05 ± 0.16 ^a^	2.28 ± 0.17 ^b,1^	2.49 ± 0.17 ^b,1^	3.41 ± 0.17 ^c,1^
Day 31 ratio	0.20 ± 0.17 ^a^	1.77 ± 0.16 ^b,1^	2.29 ± 0.16 ^c,1^	3.42 ± 0.18 ^d,1^
Enterodiol				
Day 10 ratio	0.13 ± 0.14 ^a^	1.99 ± 0.14 ^b,1^	2.93 ± 0.14 ^c,1^	3.26 ± 0.14 ^c,1^
Day 31 ratio	0.08 ± 0.14 ^a^	2.29 ± 0.14 ^b,1^	3.12 ± 0.14 ^c,1^	3.27 ± 0.14 ^c,1^
Arabinose				
Day 10 ratio	−0.60 ± 0.22 ^a,1^	−0.08 ± 0.23 ^b^	−0.17 ± 0.23 ^a,b^	0.19 ± 0.23 ^b^
Day 31 ratio	−0.46 ± 0.22 ^a,1^	−0.41 ± 0.22 ^a^	0.71 ± 0.21 ^b,1^	0.91 ± 0.23 ^b,1^
Ribulose/xylulose				
Day 10 ratio	−0.65 ± 0.30 ^a,1^	−0.36 ± 0.31 ^a,b^	−0.54 ± 0.31 ^a^	0.40 ± 0.31 ^b^
Day 31 ratio	−0.45 ± 0.32 ^a^	−0.28 ± 0.30 ^a^	0.87 ± 0.29 ^b,1^	0.96 ± 0.33 ^b,1^

^1^ Significantly different (*p* < 0.05) from baseline (Day 0). Different superscripted letters represent significant differences within a row (*p* < 0.05).

## Data Availability

Data are available in the paper and its supplemental files or by request from the corresponding author.

## Supplementary Materials

The following are available online at https://www.mdpi.com/article/10.3390/ani12131654/s1, Table S1: Formulations of the foods used in this study. Table S2: Polyphenol intakes specifically derived from the fiber bundle inclusion in the foods consumed by cats in this study.
