# Supplementary material for: Feeding Fiber-Bound Polyphenol Ingredients at Different Levels Modulates Colonic Postbiotics to Improve Gut Health in Cats
Source: Animals (Basel). 2022 Jun 27;12(13):1654. doi: 10.3390/ani12131654 (PMC9265048; doi:10.3390/ani12131654)
Supplement: Supplementary file 1 [file animals-12-01654-s001.zip › Feline ActivBiome titration ms_Table S1.pdf]

animals

MDPI

Table S1. Formulations of the foods used in this study.

	Fiber bundle percentage in food			
**Ingredients, %**	**Control**	**1%**	**2%**	**4%**
Barley, pearled, cracked	19.74	19.74	17.33	16.75
Corn, gluten, meal	19.74	19.74	17.33	16.75
Chicken, dried, 10% ash	13.46	13.46	15.37	19.40
Chicken fat, acidified	11.23	11.23	11.01	10.78
Chicken meal	8.00	8.00	8.15	6.00
Rice, brewers	7.80	6.80	10.00	8.00
Corn, yellow, whole	6.20	6.20	5.06	4.54
Egg, dried, pelleted	6.00	6.00	6.00	6.00
Palatant, LMP1	2.50	2.50	2.50	2.50
Lactic acid, blend, 84%	1.20	1.20	1.20	1.20
Potassium chloride	1.00	1.00	1.00	1.00
Calcium sulfate	0.75	0.75	0.70	0.70
Methionine, dL	0.50	0.50	0.50	0.50
Vitamin premix	0.60	0.60	0.60	0.60
Choline chloride, liquid, 70%	0.37	0.37	0.40	0.43
Taurine	0.35	0.35	0.36	0.35
L-Lysine hydrochloride	0.26	0.26	0.20	0.20
Sodium chloride, iodized	0.16	0.16	0.14	0.14
Mineral, premix, 2305	0.09	0.08	0.10	0.10
Vitamin E, adsorbate, 50%	0.03	0.04	0.04	0.04
Magnesium oxide	0.02	0.02	0.02	0.01
Pecan shells, ground	0	0.43	0.85	1.70
Flax seed, whole brown	0	0.18	0.37	0.74
Beet, pulp, pelleted	0	0.18	0.35	0.71
Citrus pulp, dried ground	0	0.15	0.30	0.61
Cranberry pomace	0	0.06	0.12	0.24
