# Supplementary material for: Feeding Fiber-Bound Polyphenol Ingredients at Different Levels Modulates Colonic Postbiotics to Improve Gut Health in Cats
Source: Animals (Basel). 2022 Jun 27;12(13):1654. doi: 10.3390/ani12131654 (PMC9265048; doi:10.3390/ani12131654)
Supplement: Supplementary file 1 [file animals-12-01654-s001.zip › Feline ActivBiome titration ms_Table S2.pdf]

animals

Table S2. Polyphenol intakes specifically derived from the fiber bundle inclusion in the foods consumed by cats in this study.

		**Fiber bundle percentage in food**		
**Intakes, mg/BW$^{0.75}$**	**Control food**	**1%**	**2%**	**4%**
Free polyphenols	0	8.73 ± 0.82	18.2 ± 2.02	35.8 ± 5.01
Bound polyphenols	0	10.7 ± 1.0	22.2 ± 2.46	43.7 ± 6.12
Total polyphenols	0	19.4 ± 1.83	40.4 ± 4.48	79.5 ± 11.1

Values are mean ± standard deviation.

BW, body weight.
